# A Multichannel Calorimetric Simultaneous Assay Platform Using a Microampere Constant-Current Looped Enthalpy Sensor Array

**DOI:** 10.3390/s17020292

**Published:** 2017-02-04

**Authors:** Hsien-Chin Wei, Su-Hua Huang, Joe-Air Jiang, Yeun-Chung Lee

**Affiliations:** 1 Department of Bio-Industrial Mechatronics Engineering, National Taiwan University, Taipei 10617, Taiwan; d94631001@ntu.edu.tw (H.-C.W.); jajiang@ntu.edu.tw (J.-A.J.); 2 Department of Biotechnology, Asia University, Taichung 41354, Taiwan; shhuang@asia.edu.tw; 3 Education and Research Center for Bio-Industrial Automation, National Taiwan University, Taipei 10617, Taiwan; 4 Department of Medical Research, China Medical University Hospital, China Medical University, Taichung 40447, Taiwan

**Keywords:** multichannel calorimeter, enthalpy sensor array, adjustable microampere constant current loop, simultaneous assay, relative enzyme activity, catalase

## Abstract

Calorimetric biochemical measurements offer various advantages such as low waste, low cost, low sample consumption, short operating time, and labor-savings. Multichannel calorimeters can enhance the possibility of performing higher-throughput biochemical measurements. An enthalpy sensor (ES) array is a key device in multichannel calorimeters. Most ES arrays use Wheatstone bridge amplifiers to condition the sensor signals, but such an approach is only suitable for null detection and low resistance sensors. To overcome these limitations, we have developed a multichannel calorimetric simultaneous assay (MCSA) platform. An adjustable microampere constant-current (AMCC) source was designed for exciting the ES array using a microampere current loop measurement circuit topology. The MCSA platform comprises a measurement unit, which contains a multichannel calorimeter and an automatic simultaneous injector, and a signal processing unit, which contains multiple ES signal conditioners and a data processor. This study focused on the construction of the MCSA platform; in particular, construction of the measurement circuit and calorimeter array in a single block. The performance of the platform, including current stability, temperature sensitivity and heat sensitivity, was evaluated. The sensor response time and calorimeter constants were given. The capability of the platform to detect relative enzyme activity was also demonstrated. The experimental results show that the proposed MCSA is a flexible and powerful biochemical measurement device with higher throughput than existing alternatives.

## 1. Introduction

Catalase is a common enzyme present in nearly all living organisms exposed to oxygen, and it is a crucial enzyme in protecting cells from oxidative damage engendered by reactive oxygen species. Catalase activity has been demonstrated to be involved in the development of human type 2 diabetes [1,2]. Erythrocyte catalase is the principal regulator of hydrogen peroxide metabolism; any inherited or acquired deficiencies in erythrocyte catalase may thus cause the hydrogen peroxide concentration in the body to increase, which can have both toxic and physiological effects. Blood catalase activity is generally measured using a clinical spectrophotometric assay. The blood catalase activity of a reference range of 1753 patients was discovered to be 80.3–146.3 units/µL [3]. The activity assay of this enzyme and other enzymes is usually challenging and time-consuming to quantify using chemical analysis [3,4].

Catalase catalyzes the decomposition of hydrogen peroxide into oxygen and water and generates a molar enthalpy of −98.2 kJ/mol [5], given by the expression:

(1) $$H_{2}O_{2}\overset{\mathrm{Catalase}}{\to }\frac{1}{2}O_{2}+H_{2}O+\left(-98.2\frac{\mathrm{kJ}}{\mathrm{mol}}\right)$$

Calorimetric measurements are principally enthalpy measurements. The heat change caused by a biochemical or physical reaction is measured using the temperature change of a solution in a calorimeter. Microcalorimeters are currently used to detect and characterize substrate concentrations, enzyme activity, and protein-ligand structures [6]. The enthalpy sensor (ES) array is a crucial device in microcalorimeters used in the physical, chemical, and biochemical fields, especially for the high-throughput assays required in modern science. Weber and Salemme reported that high-throughput calorimeters can be incorporated into the drug discovery process [7]. Enthalpy can be measured in calorimeters using three quantities: temperature change, power compensation, and heat conduction. The enthalpy of protein-small molecular and protein-protein interactions has been detected to be between −90.8 and 7.95 kJ/mol in some studies [6,7]. An enthalpy sensor with microjoule sensitivity can be used to calorimetrically measure micromoles of protein.

In recent years, microfabricated enthalpy arrays have been developed using *n*^+^ amorphous silicon [8] and vanadium oxide [9,10] thermistors for detecting the heat involved in biochemical reactions. The major merits of these enthalpy arrays are that they are very small and have a high heat sensitivity level for calorimetric assays. Each detector of these enthalpy arrays applies a Wheatstone bridge, and a reaction volume of only 500 nL (two 250 nL drops) is required. The enthalpy arrays employ an on-chip electrostatically merging and mixing mechanism using voltages of 100 and 180 V to commence the calorimetric reaction. The temperature coefficients of resistance (TCR) and noise equivalent temperature differences of *n*^+^ amorphous silicon and vanadium oxide thermistor were determined to be 2.8%/°C and 2.7%/°C and 50–100 and 30 μK root mean square (rms), respectively. The detectable temperature change, detectable heat, and thermal dissipation times were reported to be 500 and 30 μK, approximately 1 and 46 µJ, and approximately 1.3 and 2.0 s, respectively. A Deerac spot-on liquid handling system was used to deposit drops on these enthalpy arrays. Another chip calorimeter using a multichannel three-dimensional thermopile for basic applications was reported [11]. This chip calorimeter was evaluated using Joule heating and TRIS/HCl reactions, and its temperature sensitivity and heat power sensitivity were determined to be 78 μV/K and 63.2 mV/W, respectively.

Microcalorimeters used for biochemical reactions need to be able to measure temperature changes ranging from tens of microKelvins and hundreds of milliKelvins. A thermopile-based chip calorimeter cannot measure real temperatures but only the change in voltage that occurs with a change in heat. Such enthalpy arrays and chip calorimeters [8,9,10,11] involve a chip with a thermal sensor array, but are not true multichannel calorimeters. These devices use a commercial nanovoltmeter or Wheatstone bridge amplifier to measure the output voltage of a thermopile or thermistor array.

The Wheatstone bridge circuit’s ability to measure small changes in electrical resistance has been long proven, especially when a strain gauge or a Pt-100 probe is employed [12]. Therefore, the Wheatstone bridge circuit has been used in enthalpy arrays. However, this is only suitable for null detection and low-resistance sensors, as reported by Anderson [13].

The principle of thermodynamics states that the heat evolved during a biochemical reaction at constant pressure is equal to the change in enthalpy. Heat is a form of energy that causes temperature changes and can be detected by various thermal sensors such as thermocouples, negative temperature coefficient (NTC) thermistors, platinum resistance temperature detectors, and semiconductor sensors [14]. For the detection of microJoule-level heat, an NTC thermistor appears to be a suitable choice because of its high sensitivity, short response time, small size, and low manufacturing costs. However, the relationship between the temperature and resistance of an NTC thermistor is nonlinear. To achieve high sensing performance, it is necessary to consider array-excited methods, nonlinear calibration equations, and signal-amplifying methods when using the signal conditioner of an NTC thermistor-based ES array.

Previously, our group developed a prototype single-channel calorimetric assay device using a single NTC-thermistor-based ES, 200-μL acrylic reaction cell and a homemade half-bridge Wheatstone amplifier [15]. This simple calorimeter had a calibrated temperature accuracy of ± 0.001 °C and a residual H_2_O_2_ detection limit of 53 ppm. The relationship between temperature change and H_2_O_2_ concentration was obtained as 0.0079 °C/mM when using catalase as catalyst.

To yield higher accuracy with a smaller amount excitation compared to using the Wheatstone bridge while accomplishing additional measurement functions, we have designed an adjustable microampere constant current (MACC) looped-type source such that multichannel ES arrays can be implemented in MCSA platform. Such structures have not been reported by others. The contributions and aims of this work are: (1) design a novel seven channel microcalorimeter array and a new detachable reaction vessel to perform multichannel simultaneous calorimetric assays; (2) design a novel adjustable microampere constant current source to excite a seven NTC-thermistor-based ES array in a single current loop to overcome the limitations of Wheatstone bridges; (3) design a novel dual-work mode multichannel signal processing circuit for ES arrays to support high precision temperature measurements and a voltage differential measurement mode; (4) preliminarily evaluate relative enzyme activity using multichannel simultaneous calorimetric measurements.

## 2. System Design

In this study, we designed a novel MCSA platform for simultaneously detecting multiple enzyme activities in a biochemical assay. To the best of our knowledge, no similar design has been previously reported. The MCSA platform comprises a measurement unit and a signal processing unit.

### 2.1. Measurement Unit

**Figure 1:**
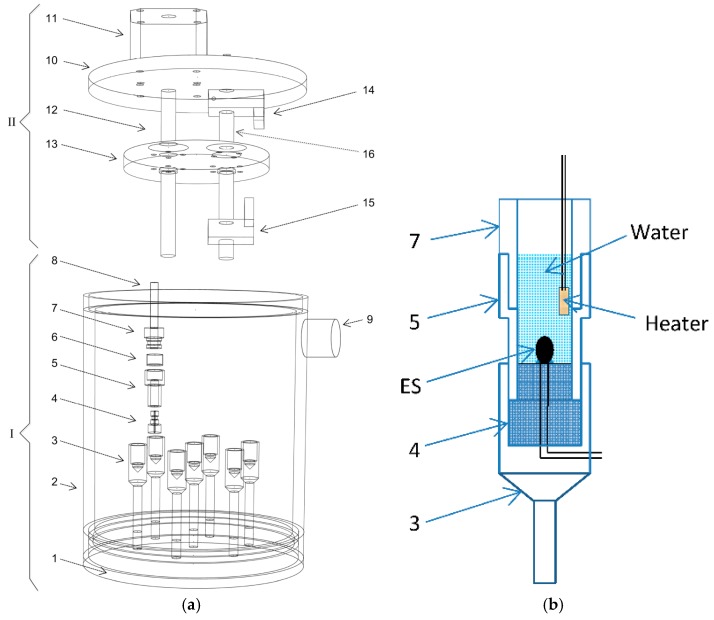
(**a**) The three-dimensional exploded view of the measurement unit of the proposed multichannel calorimetric simultaneous assay platform; only one of seven detachable reaction vessels is displayed; (**b**) Detailed arrangement of sensor plugs and chip heater in detachable reaction vessel during electrical calibration.

Figure 1a presents a complete three-dimensional exploded view of the interior and exterior of the MCSA platform measurement unit. The components of the platform are outlined as : (1) bottom of the unit, composed of a highly thermally conductive metal (Al); (2) Al wall, (3) Al calorimeter holder, (4) sensor plug (4.9 mm diameter, 23 mm high), composed of polytetra-fluoroethene (PTFE), a poorly thermally conductive polymer; (5) PTFE down cell of the calorimetric vessel (4.9 mm inner diameter, 12 mm outer diameter, 12 mm high); (6) polyethylene (PE) isolating film (10 µm thick), (7) PTFE up cell of the calorimetric vessel (4.9 mm inner diameter, 12 mm outer diameter, 22 mm high); (8) polypropylene (PP) pipe knife with 45° blade; (9) sensors’ wire connectors; (10) top cover made of another poorly conductive polymer, polymethylmethacrylate (PMMA); (11) stepping motor (ZKM2283-02TE2, Zi Sheng Motion Technologies Inc., Taichung, Taiwan) for injection; (12) injecting actuator of the stepping motor screw shaft; (13) PMMA simultaneously injecting plate; (14) up-position limit switch of the injecting plate; (15) down-position limit switch of the injecting plate; and (16) slip shaft (PSFGN-L100-F20-P4, Misumi Taiwan Cor., Taipei, Taiwan) for the injecting plate, linear bearing, and limit switches. The measurement unit consists of a multichannel calorimeter (I) and an automatic multichannel simultaneous injector (II). Figure 1b displays the detailed arrangement of the sensor plug and chip heater in a detachable reaction vessel when electrical calibration.

The calorimeter includes seven channels, where six channels are used for biochemical reactions and one channel serves as a thermal reference. All ES arrays are connected through a wire connector and wired by seven pairs of 90-cm-length insulated Cu cable (core resistance: 0.169 Ω ± 0.001 Ω per 90 cm) to the AMCC looped-type source in the signal processing unit. A plate-type automatic injector was designed to execute simultaneous multichannel injection in the measurement unit of the MCSA platform. The operation of the injector is trigged by a manual/automatic injection controller (not shown), consisting of a motor driver, a microcontroller, and a power supplier.

A thermal sink was required to stabilize the temperature of the microcalorimeter array block. Accordingly, our design involves a circulation water bath that comprises an immersed water pump, a thermoelectric cooling (TEC) device, a controllable pulse width modulation (PWM) power regulator, a 12 VDC power supplier, a thermostatic water tank with cover, and a proportional-integral-derivative (PID) temperature controller. The water bath maintains the temperature of the microcalorimeter array block at 298.13 ± 0.1 K. The baseline temperature fluctuation of the detachable thermal reference reaction vessels was determined to be approximately ±0.002 K in within an hour.

### 2.2. Signal Processing Unit

**Figure 2:**
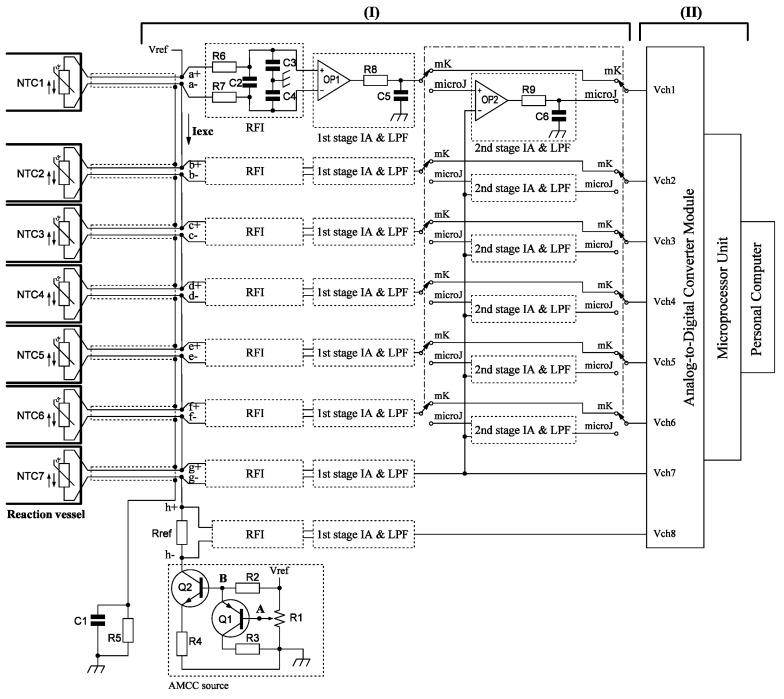
Schematic diagram of the signal processing unit including multiple ES signal conditioners (I) and a data processor (II).

The signal processing unit is mainly consists of a sensor signal conditioner (I) and a data processor (II) as illustrated in Figure 2.

The sensor signal conditioner comprises a sensor array, AMCC source, radio frequency (RF) interferometer, and 10-fold-gain instrument amplifier (IA) with passive low-pass-filter in the first-and second-stage circuits. A pair switch enables switching between “mK” and “microJ” work mode. The “mK” work mode measures of milliKelvin temperature changes, whereas the “microJ” work mode measures microJoule voltage changes.

The sensor array consists of seven NTC-thermistor-based ESs and one current sensor. The sensor array is connected in series with the AMCC source in a loop circuit. Seven commercial available interchangeable ultraprecision NTC-thermistor-based ESs, (PR103J2, accuracy ±0.05 K, R_298.13K_ = 10 kΩ, β = 3892 K, Dissipation Constant [DC] = 1 mW/K; US Sensor Corp., Orange, CA, USA), are installed in seven sensor plug (Figure 1) of the multichannel microcalorimeter array and connected separately by seven 90-cm-length dual-core-insulated Cu cables to the terminal of handmade circuit board. The current sensor is a highly accurate low-temperature coefficient resistor (10 kΩ, 0.01%, TC5; Caddock Electronics Inc., Roseburg, OR, USA). Installed directly on terminal of hand-welded circuit board, it serves as a standard resistor to measure the constant current flowing in the sensor loop in real time.

In this study, we designed a novel AMCC source to excite the sensor array. A transistor NPN (Q2) works as an open-collector constant-current source that is compensated by a single transistor PNP (Q1). The circuit is presented in Figure 2, and the collector current is given by

(2) $$I_{CQ2}=\beta_{Q2}\times I_{BQ2}$$

The current *I_C_*_Q2_ is related to the base current of Q1 as

(3) $$I_{CQ2}=\beta_{Q2}\times [I_{R2}-(\beta_{Q1}+1)I_{BQ1}],$$

where *I_B_*_Q1_ is determined by

(4) $$I_{BQ1}=\frac{V^{+}-V_{EB1}-V_{BQ1}}{R_{BQ1}}$$

*I_R_*_2_ is the current of R2, *V*^+^ is the power of +5 V dc, *V*_*EB*1_ is the potential between the emitter and the base of Q1, *V*_*BQ*1_ is the base bias voltage of Q1 and *R*_*BQ*1_ is the base resistance of Q1. According to Equations (2) and (3), *I*_*CQ*2_ is high when the base bias voltage of Q1 (*V*_*BQ*1_) is high, because of *I*_*QB*1_ is low. When *V*_*BQ*1_ is kept constant, *I*_*CQ*2_ is constant.

To reduce RF noise, an RF interferometer (RFI) was used in front of the inputs of differential IA by using a differential low-pass filter [16]. In our design, the −3 dB differential bandwidth and common-mode bandwidth were determined to be 7.5 and 159.1 Hz, respectively.

The data processor consists of an ADC module and a microprocessor unit (MPU).

All conditioned sensor signals are sent to the ADC module (LTC2418CGN; Linear Technology Company, Milpitas, CA, USA) to digitize the eight amplified sensor signals. In our study, the reference voltage (V_REF_) was supplied from VCC source (+5 V dc). The full-scale bipolar input range was from −0.5 to +0.5 V_REF_.

A user-programmable single-chip MPU (Linduino One; Linear Technology Company, Milpitas, CA, USA) module was used to digitize the amplified multiple ES array analog signals, perform digital voltage-to-temperature conversion, and transmit data to a personal computer. Windows-based data-logging software (MakerPlot; Selmaware Inc., Rocklin, CA, USA) was used to monitor and record data from the data processing unit. Microsoft Excel was used to retrieve data and perform curve-fitting.

## 3. Experiments

### 3.1. Performance Evaluation of AMCC Source

To minimize the self-heating effect of the NTC-thermistor-based ES array to a level of 1 µW, we self-designed an AMCC source. The 1-µW level guaranteed that the temperature change caused by the self-heating effect of the sensor would be less than 1 mK. The power of self-heating (P_SH_) is defined as the product by the square of the excited current (I_exc_) and the resistance of the sensor (R_sensor_) at a working temperature 298.15 K: P_sh_ = I_exc_^2^ × R_sensor_. The self-heating-induced temperature change (T_SH_) is defined as the division of the power of self-heating by the dissipation constant (DC): T_SH_ = P_SH_/DC. When the AMCC source was adjusted by less than 8 µA, the T_SH_ was controlled at less than 640 µK.

The accuracy of the AMCC source was evaluated by the following tests. The correlation between the input voltage and output current of the AMCC source in the range 0–50 µA was determined through real measurements and software simulation (LTspice IV; Linear Technology Company, Milpitas, CA, USA). Tests of the static and dynamic stability level of the AMCC source were also performed. The static stability evaluation was conducted by inserting current probes between the current sensor (Rref) and NPN collector terminal (Q2). A 6.5-digit-precision multifunction meter (DMM-4040; Tektronix, Beaverton, OR, USA) in the range of 3–10 µA for every µA was employed in the static stability test. The dynamic stability evaluation was conducted by allowing the MCSA platform to measure real-time data in the range of 2–8 µA for every µA over a period of 1 h. Microsoft Excel was used to perform the statistical analysis of the dynamic stability test.

### 3.2. Electrical Calibration of the MCSA Platform

Twelve miniature microwatt-level chip heaters were used to electrically calibrate the proposed MCSA platform. A 1.25 V alkaline battery (AA size; Panasonic, Osaka, Japan) was used as the power source of the heat pulse generator. To improve control precision during a heat pulse, a digital timer (H5CX-A-N, Omron, Osaka, Japan) was used and set at 0.01-s resolution.

During each electrical calibration, six miniature chip heaters were individually immersed in the six calorimeter reaction vessels. The vessels were filled with 200 µL of pure water and ESs were installed at the bottom-center of each vessel (Figure 1). Each heater chip was soldered with a pair of AWG30 silver-coated copper wires (R30-1000; OK Industrial, Ishpeming, MI, USA) and connected to the timer.

The Joule heating for the electrical calibration process was controlled using a manual trigger switch. When the timer relay was switched, the battery current flowed through all six miniature chip heaters and produced a heat pulse in each calorimeter reaction vessel.

To test the sensor’s application range, six miniature heaters with heating rates (resistance value) of 78 (20 kΩ), 156 (10 kΩ), 313 (4.99 kΩ), 781 (2.0 kΩ), 1562 (1.0 kΩ), and 3110 (499 Ω) µW (±1%) were used in the electrical calibration process. The sensor array was excited with a current of 8 µA. The potential difference between two terminals of each sensor was amplified with the first-stage 10-fold-gain IA (i.e., using the “mK” mode). The baseline noise levels were measured after initial thermal equilibrium was attained, and statistical analysis was performed, calculating the maximum, minimum, mean, and standard deviation in Kalvin.

To test the sensors’ precision of measurement, miniature heaters with heating rates (resistance value) of 78 (20 kΩ), 97 (16 kΩ), 104 (15 kΩ), 111 (14 kΩ), 120 (13 kΩ), and 130 (12 kΩ) µW (±1%) were used in the electrical calibration process. The sensor array was excited with currents of 3, 5, and 8 µA, and the potential difference between each sensor was amplified with two-stage 10-fold-gain IA (i.e., using the “microJ” mode). The baseline noise levels were measured after initial thermal equilibrium was attained, and statistical analysis was performed, calculating the maximum, minimum, mean, standard deviation, and peak-to-peak voltage in µV and equivalent Kelvin.

### 3.3. Calorimetric Enzyme Activity Assay

The evolution of heat is one of the general features associated with exothermic bio-chemical reaction. The total heat evolution under adiabatic conditions is proportional to the molar enthalpy change (–Δ*H*) and to the total number of product molecules (*n_p_*) created in the reaction. The resulting temperature change (Δ*T*) is inversely proportional to the total heat capacity of the system (*C_SYS_*) including the heat capacity of the solvent. This relationship can be described by

(5) $$\Delta T=(n_{p})(-\Delta H)/C_{SYS}$$

The change in temperature can then be measured using the ES in the calorimeter to determine system’s heat capacity, substrate concentration, protein concentration, and relative enzyme activity during biochemical reactions.

In this study, we demonstrated the performance of the proposed MCSA platform by observing one exothermic enzyme reaction and measuring its relative activity.

Purified catalase (CAT), from bovine liver (C3155; Sigma-Aldrich Cor., St. Louis, MO, USA), was used instead human blood. Relative catalase activity was examined using a modified Nelson’s method before the calorimetric assay. Briefly, phosphate buffer (0.05 mol/L at pH 7.0) was prepared and used for dilution and reactions. Hydrogen peroxide (0.02 mol/L) was prepared and used as substrate. A typical test used 120 µL of hydrogen peroxide in each calorimetric vessel down cell; catalase assay solution was diluted to 1/1, 1/2, 1/3, 1/4, 1/5 and 1/6 fold and filled into the up cell during an assay.

The real concentration of hydrogen peroxide substrate solution was calibrated using a spectrophotometer (USB4000, Ocean Optics, Inc., Dunedin, FL, USA) operating at λ = 240 nm; the concentration was determined every 10 s for 5 min at 25 °C. The actual H_2_O_2_ concentration was calculated by using Beer’s Law (ε^mM^ = 0.0436) [17]:

(6) $$[H_{2}O_{2}](\mathrm{mM})=A_{240}/0.0436,$$

where *A*_240_ is the absorbance of substrate solution at 240 nm.

The actual activity of the diluted catalase solution was calibrated according to the rate of decomposition of hydrogen peroxide, which is proportional to the reduction of the absorbance of hydrogen peroxide at 240 nm. Hydrogen peroxide concentrations were then calculated using the measured absorbance of the solution at a wavelength of 240 nm, with the optical path length being 1 cm and the molar extinction coefficient was 0.0436 mM/cm [18]. Catalase activity was calculated by

(7) $$\mathrm{units}/\mathrm{mL}=\frac{[\Delta A_{240}/\min(blank)-\Delta A_{240}/\min(cat)]\times d\times l}{0.0436\times V}$$

where Δ*A*_240_/min(*blank*) is the slope of reduction rate of the blank H_2_O_2_ solution at 240 nm per min, Δ*A*_240_/min(*cat*) is the slope of reduction rate of H_2_O_2_ and catalase mixing solution at 240 nm per min, *d* is dilution of the catalase solution, *l* is optical path length in cm, *V* is the reaction solution sample volume (mL), and 0.0436 is the molar extinction coefficient of H_2_O_2_ (mM/cm). The calibrated catalase activities for calorimetric test were 0.296, 0.247, 0.198, 0.148, 0.099, and 0.049 units/µL.

## 4. Results and Discussion

### 4.1. Accuracy and Stability of AMCC Source

**Figure 3:**
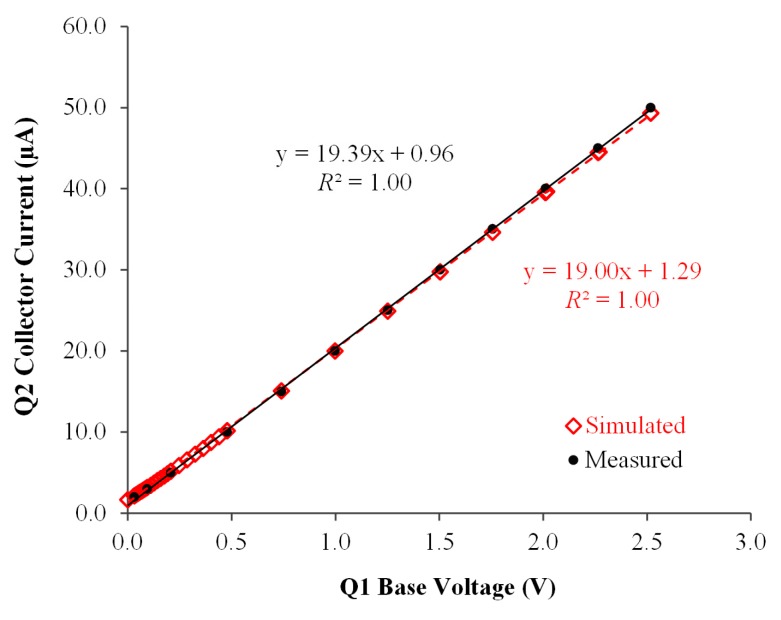
Relationship between input voltage and output current of the proposed transistors-based adjustable microampere constant-current source.

Real data were measured by using a 6.5-digit-precision multifunction meter (DMM-4040, Tektronix), whereas simulated data were obtained using LTspice IV software (Linear Technology Corporation). As illustrated in Figure 3, both the measured (black) and LTspice IV simulated (red) output currents of the proposed AMCC source were linear when the base bias voltage of Q1 (*V*_*BQ*1_) varied from 0.07 to 2.518 V. The difference in the coefficients of determination between the two regressions lines was relatively small. Such small differences are mainly caused by the insertion of current meter probes serially between the current sensor (Rref) and the NPN collector (Q2) when measuring a real current.

The accuracy and stability of the current directly affect the performance of the proposed ES array. Static stability analyses were conducted using the statistic function built into the 6.5-digit-precision multifunction meter (DMM-4040) with respect to 3000 measured data points. The results indicate that the when the output current (*I*_SET_) was adjusted from 2.998 to 10.00 µA, the overall standard deviation (*I*_SD_) of the output currents was within 1.027–1.304 nA, implying that the overall error percentage ranged from 0.03% to 0.01%.

The dynamic stability of the current output was tested over a period of 1 h. Dynamic stability analyses were performed using online real-time measurement, wherein all ES arrays included current sensor are connected in current loop and the MCSA platform was operated. The measured current data were collected using the software MakerPlot. The statistical analyses were executed using Microsoft Excel. The overall standard deviation (*I*_SD_) of the output currents were within 0.271–1.652 nA, which indicates that the overall error percentage varies from 0.01% to 0.02%, when the output current (*I*_SET_) was adjusted from 2.00 to 8.00 µA. These results indicated that the lower loop current has high stability than did the higher loop current during real-time operation.

### 4.2. Performance Evaluation of the Proposed MCSA Platform

#### 4.2.1. Electrical Calibration of Application Range Using “mK” Mode

**Figure 4:**
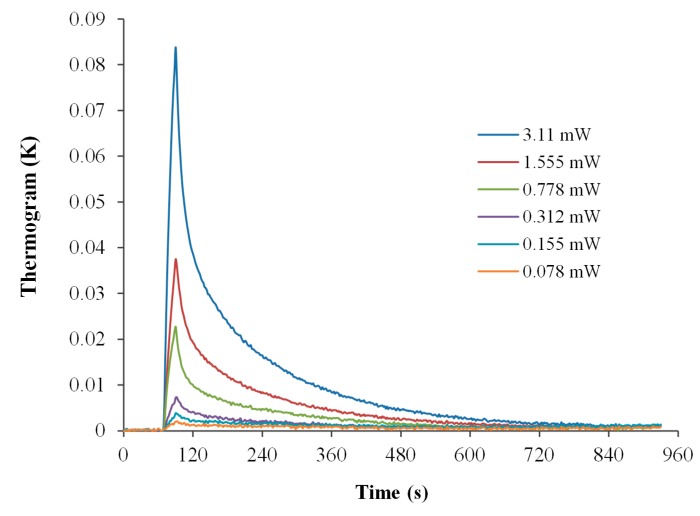
Joule heating pulses (20 s) response of the proposed multichannel calorimetric simultaneous assay platform in “mK” mode (10-fold gain IA amplifier). Baseline temperature drift rate was 4 µK/s between measurements.

The sensitivity of the proposed MCSA platform was evaluated using different Joule heating strengths. The measurement conditions used in sensitivity evaluation are outlined as follows: loop current = 8 µA; heater power voltage = 1.2468 V; heat pulse time = 20 s; gain = 10-folds; amplifier = first-stage 10-fold differential IA; heater = six miniature chip heater; power range = 0.078–3.110 mW. The temperature response curves are presented in Figure 4. The calorimeters have time constants of about 200 s. The ES array sensed heat pulses from miniature chip heaters in reaction vessels. A high peak indicates that the electrical heat pulse was terminated because the timer relay shut off the power from the battery; thus, no more heat could accumulate in the detachable reaction vessels. Since the reaction vessels were installed in aluminum holders and fixed on an aluminum bottom plate that was in thermal equilibrium with the thermal sink. Thus, the accumulated heat in the reaction vessels was quickly lost through the holder because of its high heat transfer coefficient. This caused the temperature to decrease in the reaction vessel. Finally, the total change in temperature returned to zero, indicating that the calorimeters were thermally balanced. Both the bottom plate and thermal sink were in thermal equilibrium.

The initial thermal equilibrium baseline noise levels of six microcalorimeters were statistically analyzed. The average baseline temperature noise was between 76 and 204 µK (standard deviation = 121–172 µK), equivalent to average voltage noise between 0.16 and 8.93 µV (standard deviation = 4.03–6.63 µV), respectively.

**Figure 5:**
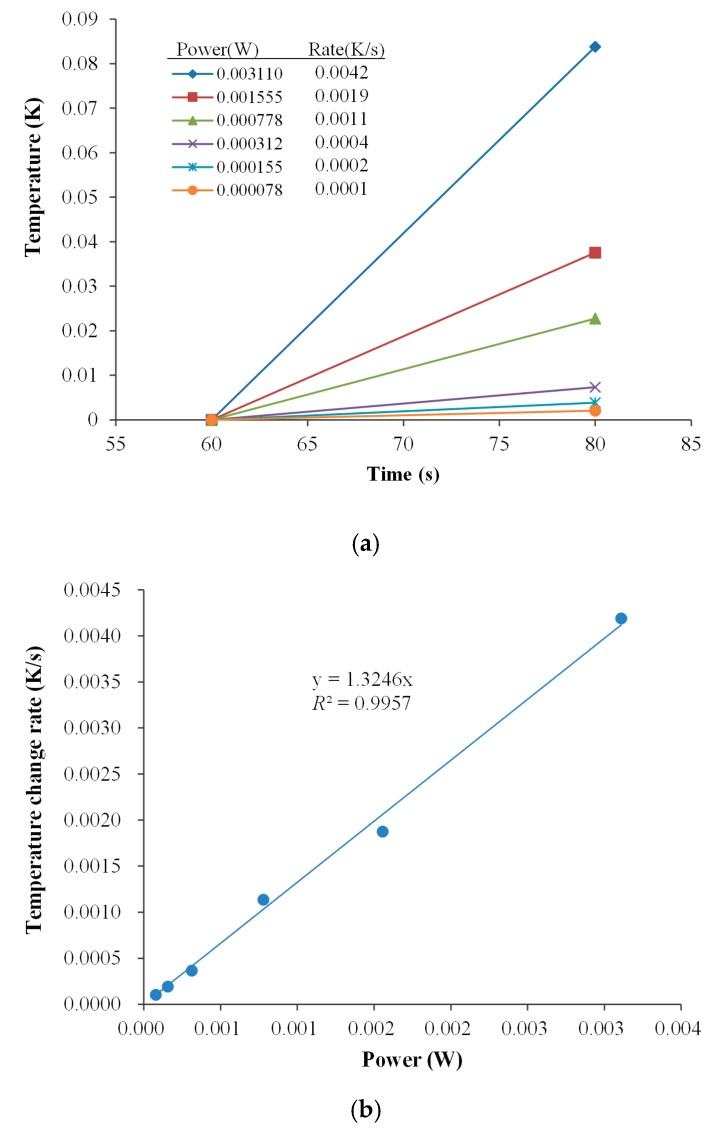
Electrical calibration of the proposed multichannel calorimetric simultaneous assay platform. (**a**) Temperature-time plot in the present of various Joule heating power of the proposed MCSA platform; (**b**) Regression curve between the temperature change rate and Joule heating power.

From the temperature–time curve (Figure 4) obtained from each individual heat pulse, a tangent was drawn for the initial part of the curve (peak high temperature change/the heat pulse time). The obtained temperature change rate (K/s) with six chip heater power settings were graphed in Figure 5a. The regression curve of the rate of temperature change versus the Joule heating power (0.000078–0.00311 W) is displayed in Figure 5b. The calibration constant was defined as the inverse of the slope of the regression, i.e., 0.76 J/K; which corresponded to temperature change sensitivity with respect to voltage, i.e., 25.84 K/V. The obtained data indicates that the detection limit of the proposed platform was 1.558 mJ in “mK” mode (loop current = 8 µA), equivalent to a temperature change rate of 0.1 mK/s.

To evaluate the percentage of heat loss preliminarily, the relationship between the input-heat and measured-heat was calculated. The measured-heat was calculated by

(8) $$\Delta H=m\times C_{P}\times \Delta T,$$

when *m* = 0.18 g is the mass of pure water in the reaction vessel, *C*_p_ = 4.186 J/g·K is the specific heat capacity of pure water, and ∆*T* is the measured temperature change (taken from Figure 4). The correlation between the calculated data and the input electrical heat was found to be linear, with a slope of 0.999. However, the calorimeter array did lose a non-negligible amount of heat. During the electrical calibration process, approximately 200 µL of pure water was added to each reaction vessel. Thus, the calorimeter array had a heat loss of at least approximately 10%.

#### 4.2.2. Sensitivity Tests Under Various Loop Currents Using “microJ” Mode

A higher exciting loop current produced higher potential in the thermistor-based sensor array. Moreover, higher amplification gain increased the sensitivity of the sensor array as well as the noise level. The system response between different loop currents were used was investigated using Joule heating in a narrow range. The measurement conditions used in sensitivity test are outlined as follows: loop current = 3, 5, and 8 µA; heater power voltage = 1.25 V; heat pulse time = 30 s; gain = 100-fold; amplifier = dual stage of 10-fold differential IA; heater = six miniature chip heater; power range = 0.000078–0.00013 W.

**Figure 6:**
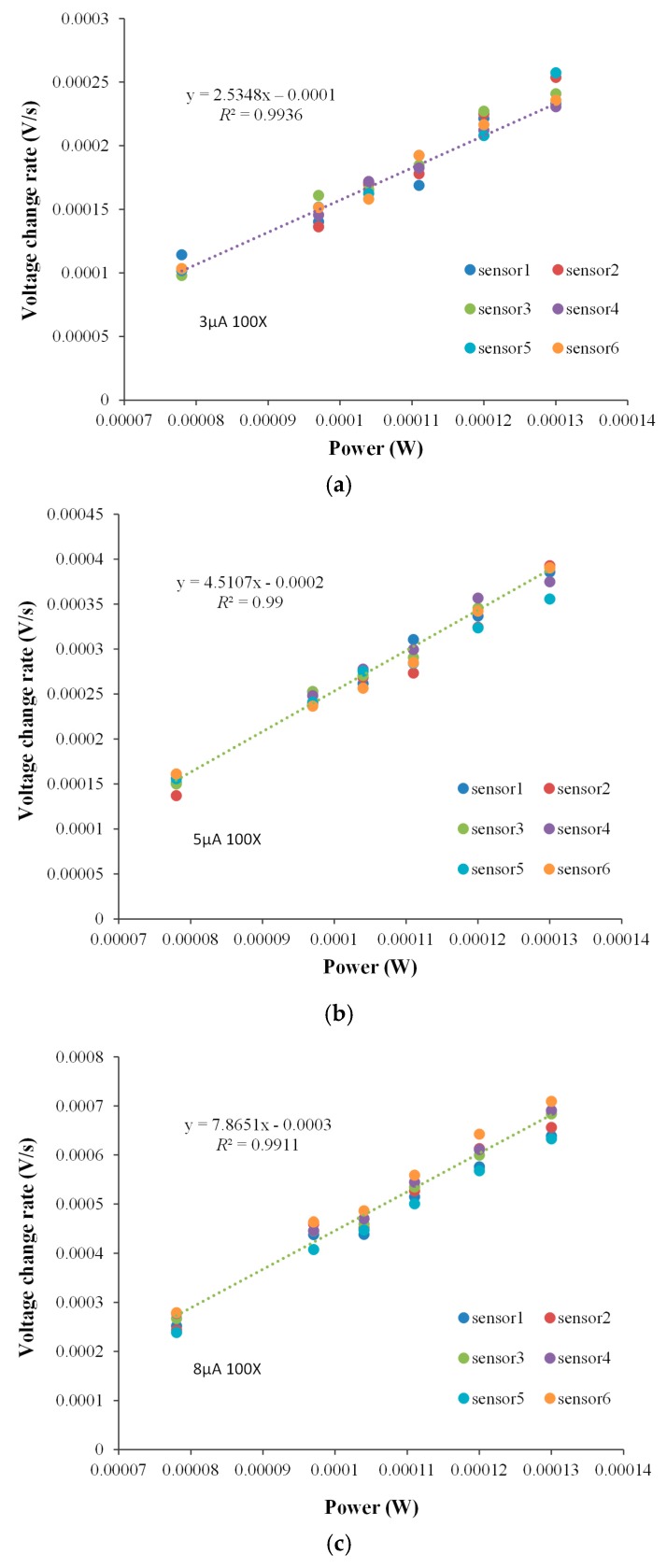
Heat pulse sensitivity for the proposed multichannel calorimetric simultaneous assay platform when various loop currents and six miniature chip heaters. Test results with respect to three loop currents: (**a**) 3 µA; (**b**) 5 µA; (**c**) 8 µA (Joule heating pulse time: 30 s).

Test results when loop currents of 3, 5, and 8 µA were used, with six runs performed for each current, and are presented in Figure 6. The correlation between the responses of the proposed MCSA platform and the power was linear. The detectable heat pulse levels of the platform when worked in “microJ” mode and the loop current of 3, 5 and 8 µA were used were 2.5348, 4.5107 and 7.8651 V/J, respectively. These data indicated that the minimum detectable heat was 1.424 µJ under 8 µA exciting current in “microJ” mode, with the standard deviation of the ADC being 11.2 µV, which corresponds to 0.00002 °C. In Figure 6, various loop currents lead to different heat pulse sensitivity. In real applications, such an arrangement would not be affected, because the experiments were used to evaluate the performances of the MCSA platform at various loop currents. The results support to use a low loop current to lower self-heating effects in the future.

**Figure 7:**
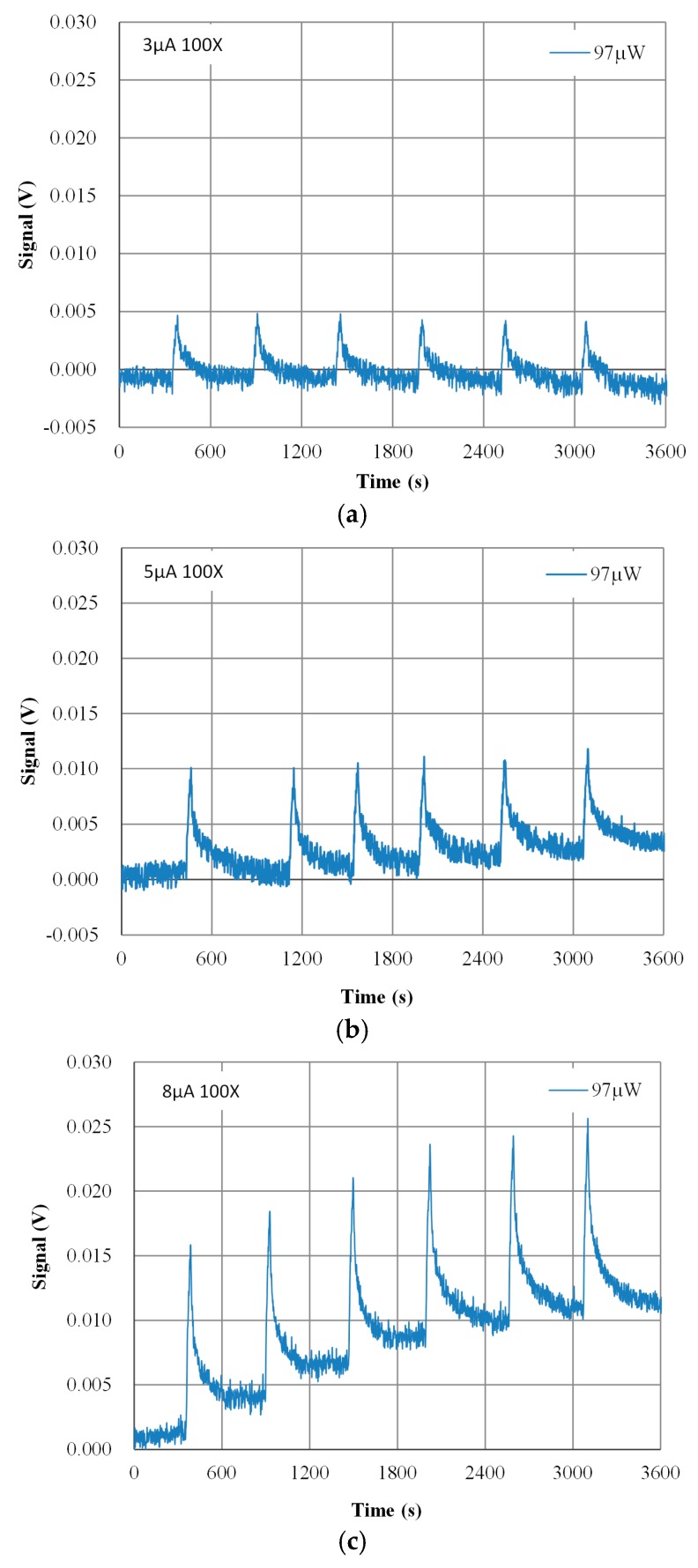
Three observed heat pulse response curves of the proposed multichannel calorimetric simultaneous assay platform under test loop currents of (**a**) 3; (**b**) 5; (**c**) 8 µA.

Figure 7 illustrates the highest baseline noise level in one of six microcalorimeter arrays. For each exciting current used, six repeat measurements with equal power resulted in similar responses. However, the noise level was substantial in the test cases of specific channel. Moreover, the baseline noise varied with the measuring time. These offsets were caused by the irregular geometric thermal distribution of the microcalorimeter array and the temperature drift of the thermal sink. This effect was particularly marked for the data in Figure 7c. In a multichannel calorimeter array design, the geometric thermal distribution should be considered carefully to minimize the long-term baseline temperature offset and drift.

The magnitude of the baseline noise was dependent on: (1) the design of the electrical circuit; (2) the layout quality of the printed circuit board (PCB); and (3) the gain of signal amplifier. The baseline noise basically was discovered to originate from the 60-Hz power line after an observation was made using a spectrum analyzer. Furthermore, the baseline noise did not decrease when the loop current was increased but the electrical noise continued in specific channels. Care must be taken when designing and laying out prototype PCBs so that baseline noise is minimized.

The details of the initial thermal-balance baseline noise between the six ESs, as illustrated in Figure 7, were statistically analyzed. The maximum noise, minimum noise, average noise, standard deviation of the average noise, peak-to-peak noise, and conversion of the baseline peak-to-peak noise from voltage into temperature were calculated. The peak-to-peak baseline noise was converted from voltage to temperature by multiplying the (p–p) by 25.84 K/V, and this was the temperature change sensitivity in the “mK” mode. The highest peak-to-peak noise was in the channel of 97-µW. When the exciting currents of 3, 5 and 8 µA were applied, the peak-to-peak noise levels were 23.83, 27.67 and 20.58 µV, respectively, equivalent to 615.77, 714.99 and 531.79 µK temperature change, respectively.

### 4.3. Catalase Activity Detection

To demonstrate the capability of the proposed MCSA platform in preliminary biochemical assay, we designed an assay experiment for detecting enzyme activities. In this assay, 2.4 µmol (20 mM × 120 µL) calibrated hydrogen peroxide solution was used as a substrate and calibrated 120-μL diluted catalase solution was used as enzyme.

The *A*_240_ of the original hydrogen peroxide substrate solution was measured as 0.428. This corresponded to a real concentration of hydrogen peroxide substrate for the calorimetric assay of 20 mM.

The catalase reaction rate, as measured by the slope of the *A*_240_ curve, was obtained as 0.1696 per min. The real initial catalase activity was calculated to be 592.66 units/mL. Six diluted catalase enzyme solutions with activities of 0.296, 0.247, 0.198, 0.148, 0.099, and 0.049 units/µL, which were calibrated by spectrophotometric method, were used for the calorimetric assay experiments.

**Figure 8:**
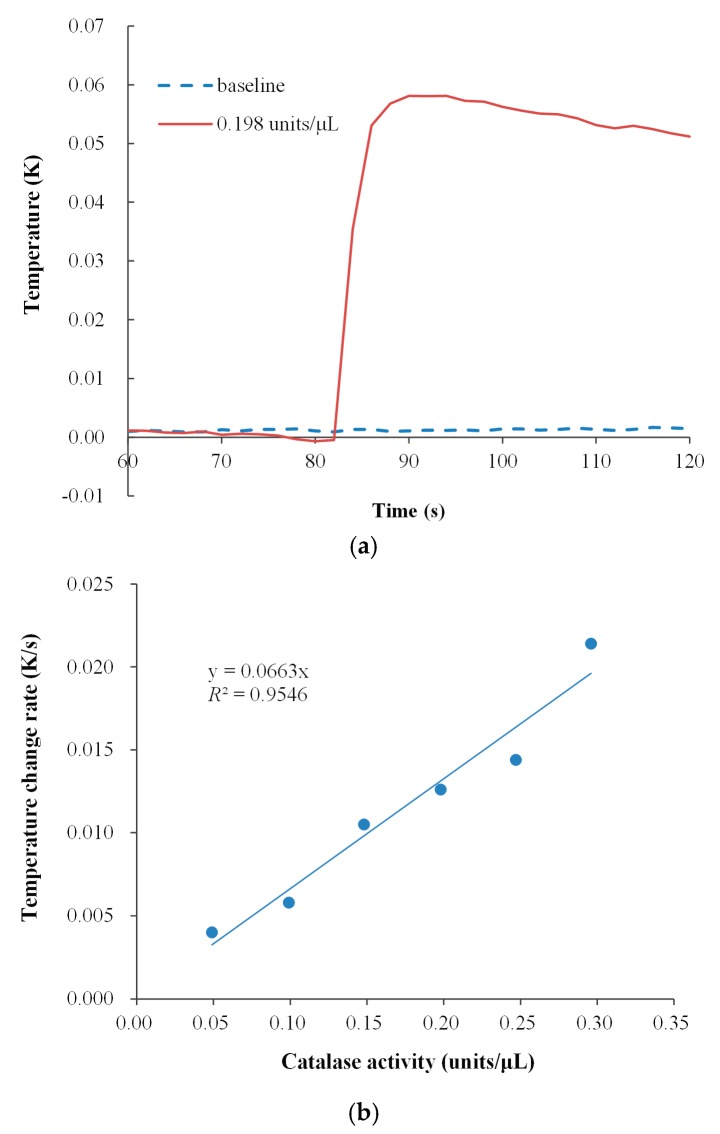
Detection of purified catalase, from bovine liver (C3155). (**a**) Thermogram example of catalase activity 0.198 units/μL; (**b**) Temperature change rates between different enzyme activities.

Figure 8 presents the preliminary results of the relative enzyme activity detection of calibrated catalase. In Figure 8a shows a thermogram of the experimental test when a solution with a relative enzyme activity of 0.198 units/µL was introduced into the lower substrate cell at 82 s. The thermal sink remained at 298.13 K as the datum line. Both the upper cell with the enzyme solution and the lower cell with the substrate one were thermally balanced with the surroundings. We did not monitor the temperature in the up cell. The thermally unbalanced upper cell solution might be a source of disturbance in the injection, and the effect might be negligible as shown in the figure. The temperature fluctuated by approximately ±0.001 K before the reaction due to the use of an accurate thermistor and a stable thermal sink, and the temperature fluctuation was minor as compared to the temperature elevation during the reaction.

Enzyme activities could be quantified on the MCSA platform by measuring the heat rate of an exothermic reaction. Assuming that the heat capacity of the sample solutions is constant, and that the reaction system is adiabatic at the very short initial stage, the heat rate could be linearly expressed in a temperature change rate. Taking the enzyme activity of 0.198 units/µL as an example. Figure 8a clearly shows that the temperature of the biocatalytic process increased linearly after the onset of the reaction. The temperature change rate of the reaction for this sample (0.198 units/µL) was estimated to be around 0.013 K/s. A similar calculation was applied to five other samples. The temperature change rates against the six enzyme activities (units/μL) are plotted in Figure 8b. The results indicate that the relationship between temperature change rate (K/s) and enzyme activity (units/μL) is linear with a slope of 0.066 K/s/unit/µL, calculated by linear regression. The reciprocal of the slope would be the measurement constant for the catalase activity on the MCSA platform.

In the report of Góth [1,3], the blood catalase activity of 1753 patients as determined by a spectrophotometric assay was 80.3–146.3 units/µL, where one unit of catalase decomposes 1 µmol of hydrogen peroxide in 1 min under these conditions and it is related to 1 mL of whole blood. When the assay is conducted with a spectrophotometric method, every test requires four preparations and four measurements, once for a sample and three times for a blank. When our proposed MCSA assay platform is used, every test requires only a 1-µL blood sample and 120 µL of buffer.

### 4.4. Discussion

The temperature resolution of the presented MCSA platform was up to ±10 µK (predicted calibration error between 20 and 30 °C = −20 µK ± 10 µK), which is improvement on the device reported in our previous work, which had a resolution of ±1000 µK (calibrated by the primary SPRT 25.5 Ω thermometer). The temperature change and detectable Joule heating of the MCSA platform were 38 mV/K and 7.865 V/J, respectively. From previous reports, the temperature and heat or power sensitivity of the Ni/Au thermocouple were 0.078 mV/K and 63.2 mV/W, respectively [11]; those of an *n*^+^ amorphous silicon (*n*^+^) thermistor were 500 µK and 0.5 K/J (TCR: 2.8%/°C), respectively [8]; and that of a vanadium oxide (VOx) thermistor was 890 K/W (TCR: 2.7%/°C) [9,10].

In recent years, microfabricated enthalpy arrays have been developed using *n*^+^ [8] and VOx [9,10] thermistors for detecting the heat involved in biochemical reactions. These enthalpy arrays are extremely small and have high heat sensitivity for calorimetric assays. However, Wheatstone bridges are used in each detector of those enthalpy arrays. Therefore, till now microfabricated enthalpy arrays still cannot provide multichannel simultaneous assay measurements. In our study, a microampere constant-current loop was employed to excite seven NTC-thermistor-based sensor arrays in the MCSA platform. The reaction volume in the *n*^+^ and VOx enthalpy array was only 500 nL (two 250 nL drops) and was dropped on the detector region, whereas two drops of 120 µL were injected separately in the up cell and down cell of the detachable reaction vessel in the MCSA platform.

An automated simultaneous plate-type injector was used in the proposed MCSA platform for mixing and initializing the calorimetric reaction, whereas an on-chip electrostatic merging and mixing mechanism using voltages of 100 and 180 V was employed in the *n*^+^ and VOx enthalpy arrays to initiate the calorimetric reaction. Both could induce a drift of the thermal signal in real operation. The TCR of the MCSA thermistor was 4.4%/°C, whereas those of the *n*^+^ and VOx thermistors were 2.8 and 2.7%/°C, respectively. In addition, the differential temperature noise levels in *n*^+^ and VOx thermistors were 50–100 and 30 μK rms, respectively; moreover, that of the MCSA platform was less than 204 μK on average. The detectable temperature change of *n*^+^ and VOx thermistors were 500 and 30 μK, respectively, whereas that of the MCSA platform was 100 μK/s. The detectable heat in the *n*^+^ and VOx thermistors and the MCSA platform in microJ were 1, 46, and 1.424 μJ, respectively. Furthermore, the thermal dissipation time in *n*^+^ and VOx thermistors were approximately 1.3 and 2.0 s, respectively, whereas that of the MCSA’s thermistor was approximately 7.72 s (response time was 1.25 s in water). An expensive Deerac spot-on liquid handling system was used to deposit the drops on the *n*^+^ and VOx enthalpy arrays, whereas a cheap manual electrical pipette was used in the MCSA platform.

## 5. Conclusions

We have developed and demonstrated a microampere constant-current-loop-excited multiple NTC-thermistor-based ES array that incorporates a multichannel microcalorimeter array in a single block. The predicted calibration temperature measurement error achieved by the MCSA platform was −20 µK ± 10 µK (between 20 and 30 °C). The newly PNP-regulated NPN-transistor-based AMCC source provided a stable current between 2 and 8 µA with an error of 0.02%. An 8-µA constant current supported the self-heating of the NTC-thermistor-based ES within a temperature level of 640 µK. When operating in “mK” mode, the proposed MCSA platform had a calibration constant of 0.76 J/K and the Joule heating heat pulse detection limit was discovered to be 1.56 mJ. When the proposed MCSA platform was operated in “microJ” mode, its detectable Joule heating heat pulse was increased to 1.424 µJ. The proposed MCSA platform was also successful in the detecting purified catalase enzyme activities. The relationship between the temperature change rate (K/s) and relative catalase activity (units/µL) was linear. Catalase as well as other enzyme activities could be estimated by measuring the temperature change rate on the MCSA platform. The experimental results show that the proposed MCSA is a flexible and powerful biochemical measurement device with higher throughput.
